# The necroptosis related gene LGALS3 can be used as a biomarker for the adverse progression from chronic HBV infection to HCC

**DOI:** 10.3389/fimmu.2023.1142319

**Published:** 2023-04-26

**Authors:** Jianming Dong, Rongzheng Zhang, Yan Xia, Xu Jiang, Kun Zhou, Jiaqi Li, Mengrui Guo, Xinyang Cao, Shuyun Zhang

**Affiliations:** ^1^ Scientific Research Center, The Second Affiliated Hospital of Harbin Medical University, Harbin, China; ^2^ Department of Parasitology, Harbin Medical University, Harbin, China; ^3^ Beidahuang Industry Group General Hospital Department of Clinical Laboratory, Harbin, China

**Keywords:** necroptosis related gene, HBV infection, hepatocellular carcinoma, immune microenvironment, prognosis

## Abstract

The number of patients with hepatocellular carcinoma (HCC) caused by hepatitis B virus (HBV) infection remains large, despite the remarkable effectiveness of antiviral drugs and vaccines for HBV in preventing and treating HBV infection. Necroptosis is closely related to the occurrence of inflammation, clearance of viral infection, and tumor progression. Presently, little is known about the changes in necroptosis-related genes in the progression from chronic HBV infection (CHI) to HBV-related hepatic fibrosis (HBV-HF) and HBV-related hepatocellular carcinoma (HBV-HCC). In this study, Cox regression analysis was performed using GSE14520 chip data and a necroptosis-related genes survival prognosis score (NRGPS) was established for HBV-HCC patients. NRGPS was constructed using three model genes (G6PD, PINK1 and LGALS3), and verified by data sequencing in the TCGA database. The HBV-HCC cell model was established by transfection of pAAV/HBV1.2_C2_, constructed by homologous recombination, into HUH7 and HEPG2 cells. The expression levels of G6PD, PINK1, and LGALS3 were detected using RT-qPCR. We further analyzed the expression of the model genes in GSE83148, GSE84044, and GSE14520 and found that LGALS3 was consistently highly expressed in CHI, high fibrosis score and high NRGPS. In addition, immune microenvironment analysis showed that LGALS3 was not only associated with the infiltration of regulatory T cells in the immune microenvironment but also with expression of CCL20 and CCR6. The expression levels of model genes, FOXP3 and CCR6, were analyzed using RT-qPCR in peripheral blood mononuclear cells of 31 hepatitis B surface antibody positive patients, 30 CHI, 21 HBV-HF, and 20 HBV-HCC. In further cell-model experiments, we analyzed the expression of CCL20 by RT-qPCR and the changes in cell proliferation and migration by CCK8 and transwell assays, respectively, in HBV-HCC cell models after LGALS3 knockdown. The findings of this study suggest that LGALS3 could be a biomarker for adverse progression following chronic HBV infection and may also be involved in the regulation of the immune microenvironment, making it a potential therapeutic target.

## 1 Introduction

Hepatitis B virus (HBV) infection is a worldwide epidemic, with an estimated 257 million patients with chronic hepatitis B worldwide. Furthermore, about 887,000 people die each year from diseases related to HBV infection, such as HBV-related hepatic fibrosis (HBV-HF) and HBV-related hepatocellular carcinoma (HBV-HCC). In China, 77% of hepatic fibrosis and 84% of hepatocellular carcinoma cases are caused by HBV (1). During chronic HBV infection (CHI) progression, the activity of the necroptotic pathway in diseased liver tissues is often enhanced (2).

Necroptosis is an important means of interacting with pathogenic microorganisms, in addition to the immune system, and it is mainly composed of receptor-interacting protein kinase 1 (RIPK1), receptor-interacting protein kinase 3 (RIPK3), and mixed lineage kinase domain-like (MLKL) protein. The changes in the conformation of the MLKL protein and translocation to the cell membrane, as well as the flow of Na^+^, Ca^2+^, and other cations increases the intracellular osmotic pressure. Furthermore, it can also directly form pore structures on the cell membrane, leading to cell destruction and release of a large number of cell contents. The leaked cell contents also form damage-related molecular patterns that allow for a massive invasion of surrounding immune cells, triggering intense inflammation (3, 4). Infected cells are eliminated through necroptosis and virus transmission is limited by the release of inflammatory mediators that promote the activation of innate and adaptive immunity (5). In this study, we used the Gene Expression Omnibus (GEO) database to develop prognostic markers of HBV-HCC composed of three necroptosis-related genes (NRGs), including LGALS3, G6PD, and PINK1. The relative-expression levels of model genes mRNA in HEPG2, HUH7, LX2, and peripheral blood mononuclear cells (PBMCs) were measured by RT-qPCR. Finally, the cancer genome atlas (TCGA) database was analyzed to verify the necroptosis-related genes survival prognosis score (NRGPS). LGALS3, a member of the galactoagglutinin family, is a vital gene in the regulation of the liver immune microenvironment (6, 7) and is significantly associated with increased risk of liver failure, cirrhosis, chronic active hepatitis B, and shorter survival time for hepatocellular carcinoma (HCC) (8, 9). Analysis of the relationship between LGALS3 and adverse disease progression after persistent HBV infection and its immune microenvironment may help understand the role of necroptosis in chronic HBV infection, which is of great significance for the prevention and treatment of adverse disease progression. Therefore, this study aimed to analyze the relationship between LGALS3 and adverse disease progression after persistent HBV infection and its immune microenvironment.

## 2 Materials and methods

**Figure 1:**
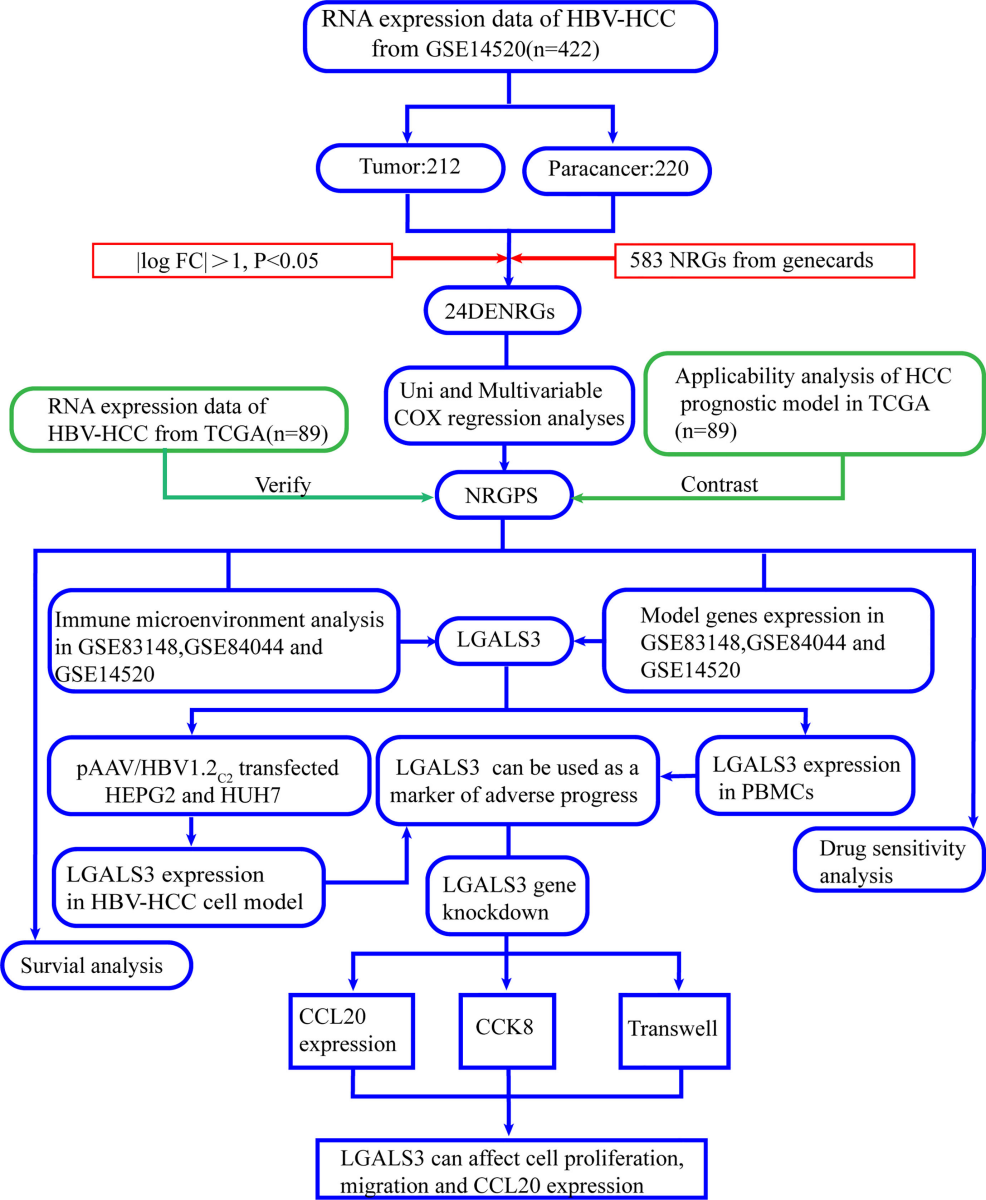
Flowchart of this study.

**Figure 1**, a flowchart, illustrates this study’s design and procedure.

### 2.1 Acquisition of data from patients with HBV infection

We downloaded the following gene expression profiles from the GEO database: GSE83148, GSE84044, and GSE14520. As the GSE14520 dataset contained two different batches of expression profile chip datasets, we selected the Affymetrix HT Human Genome U133A Array dataset, which contained the majority of patients, to avoid batch processing effects. Sequencing data from 89 HBV-HCC were obtained from TCGA. **Supplementary Table 1** shows the specific clinical information.

### 2.2 Identification of differentially expressed NRGs

Overall, 583 NRGs were obtained from GeneCards (www.genecards.org/) and their expression levels were obtained by intersection with gene expression data from GSE14520, using the “limma” R package, and with | log2 fold change (FC) | > 1 and adjusted p < 0.05 as filter conditions. Finally, differentially expressed necroptosis-related genes (DENRGs) were obtained from the tumor and paracancerous tissues. The “pheatmap,” “ggpubr,” “ggrepel,” “dplyr,” “ggplot2,” and “rcircos” R packages were used to generate heat maps, volcano maps, and chromosome pattern maps of DENRGs. Gene ontology (GO) analysis was performed using the “clusterprofiler,” “enrichment plot,” and “ggplot2” R packages.

### 2.3 Construction and validation of the NRGPS

Follow-up time ≥ 30 days was used as inclusion criteria. The “caret” R package was used to divide the 212 HBV-HCC patients randomly into the training and test sets in a ratio of 6:4. **Table 1** presents the specific clinical information for the training and test sets. Univariable Cox regression analysis was performed for 24 DENRGs in the training set using “survival” and “survminer” R packages to screen for DENRGs associated with prognosis in the training set. Subsequently, based on the survival information of the samples, including survival time and survival state, combined with the expression values of prognostic related genes in each sample, through multivariable Cox regression analysis and stepwise regression algorithm, the gene combination with the greatest impact on survival was found, combined with the corresponding regression coefficient to construct NRGPS, the calculation formula is as follows:

**Table 1:** There was no significant difference between the two groups of clinical phenotypes.

Parameters	Training set	Test set	P value
N	129	83	
Age >55 years (%)	29.46	32.53	0.636
Gender, male (%)	88.37	83.13	0.279
TNM stage			0.236
I	53	36	
II	46	30	
III	30	17	
OS time, median (IQR)	4.33 (1.5–4.82)	4.22 (1.48–4.71)	0.482

OS, overall survival; IQR, interquartile range.


$$\text{NRGPS}=\sum_{i=1}^{N}\left(Exp(gene_{i})\times Coef(gene_{i})\right)$$

Kaplan–Meier curve analysis was performed using “survival” and “survminer” R packages to determine the survival rate of high and low NRGPS groups. Time-dependent receiver operating characteristic curve analysis was performed using “survivalROC” R package to evaluate the prognostic value of survival indicators. Moreover, “Rtsne” package principal component analysis was used to reduce the dimensionality of the multivariable data in the NRGPS, and the data was visualized. Univariable and multivariable Cox regression analyses were conducted to evaluate the independent prognostic value of the NRGPS. Additionally, “c2.cp.kegg.v7.5.1. symbols.gmt” was used as the reference file, and Gene Set Enrichment Analysis (GSEA) was performed using “limma,” “GSEABase,” “GSVA”, and “pheatmap”. Finally, the potential biological processes and risk paths differing between the high and low NRGPS in the GSE14520 cohort were visualized. A nomogram based on NRGPS was developed using the “rms” R package to predict the prognosis of patients with HBV-HCC. The TCGA dataset was used as an external validation of NRGPS.

### 2.4 Prognostic analysis of existing prognostic models of necroptosis-related genes in HCC

We collected several studies (10–15) on the construction of prognostic models of HCC patients using NRGs, and conducted a Kaplan–Meier curve analysis of 89 patients with HBV-HCC in the TCGA database using the prognostic models in the literature to determine whether the prognostic models in the literature are suitable for HBV-HCC.

### 2.5 Drug sensitivity analysis

Differences in drug sensitivity between high and low NRGPS groups were analyzed using “ggplot2” and “pRRophetic” R packages in the TCGA cohort. A Ridge regression model was constructed using the pRRophetic algorithm based on the genomics of drug sensitivity in cancer cell line and TCGA gene expression profiles to calculate the difference in IC50 of chemotherapy drugs between the high and low NRGPS groups. Additionally, we downloaded the drug structures using the PubChem database (https://pubchem.ncbi.nlm.nih.gov/).

### 2.6 Immune cell infiltration analysis and functional analysis

In GSE83148, GSE84044, GSE14520, and TCGA cohorts, single-sample gene set enrichment analysis (ssGSEA) was conducted using the “GSVA” R package to analyze related immune cells and immune pathways in the microenvironment and predict the degree of immune cell infiltration. Furthermore, “reshape2,” “limma,” “tidyverse,” “corrplot,” and “ggplot2” R packages were used to analyze and map the correlation of modeling genes with chemokine receptors and ligands, immune cellsand function.

### 2.7 Clinical specimen collection

From February 2022 to August 2022, 1 mL of EDTA anticoagulant peripheral blood was collected from 71 outpatients at the Second Affiliated Hospital of Harbin Medical University as an experimental specimen, including 30 cases of CHI, 21 cases of HBV-HF, and 20 cases of HBV-HCC. The EDTA anticoagulant peripheral blood of 31 healthy patients with hepatitis B surface antibody positive (HBsAb+) was used as the control group. The inclusion criteria are shown in **Supplementary Table 2**. The clinical information is shown in **Supplementary Table 3**. Human whole blood mononuclear cell isolation solution was used to extract mononuclear cells and, after addition of 1 mL Seven RNAkey™ Reagent (SM129-02, Seven, China), samples were frozen at -80°C and stored until further use.

### 2.8 Construction of HBV-HCC cell model

Based on GenBank: JQ688404.1 Whole genome of HBV C2 subtype and 1.2 fold gene sequence of pAAV/HBV1.2 plasmid constructed by Huang et al. (16): NT140-NT3182/1-NT1987 using pAAV-MCS as carrier, pAAV/HBV1.2_C2_ recombinants were constructed by gene synthesis and homologous recombination. The process was completed by Beijing Liuhe Huada Genomics Technology Co., LTD. pAAV-MCS carrier was purchased from Harbin Suit Biotechnology Co., Ltd. A high-purity Midiprep Kit (ZP104-1, Zoman, China) was used to extract the plasmid. Moreover, HEPG2 and HUH7 cells were purchased from Wuhan Procell Life Technology Co., Ltd.; LX-2 cells were purchased from Shanghai Gaining Biotechnology Co. Ltd. Additionally, HEPG2, HUH7, and LX-2 were cultured in Dulbecco’s modified Eagle medium (DMEM) high-glucose medium (D6429, Sigma, USA) with 10% fetal bovine serum (11011-8611, Every Green, China) and 5% CO_2_ at 37°C. The pAAV/HBV1.2_C2_ and pAAV-MCS empty vectors were transfected into HEPG2 and HUH7 cells using a liposome transfection reagent (C0533, Beyotime, China), and co-transfected with the pmaxGFP plasmid. HUH7^#^ and HEPG2^#^ denote the HUH7 and HEPG2 cell lines after transfection with pAAV/HBV1.2_C2_. The supernatants were collected after 48 h. HBsAg expression in the supernatant of the cells transfected with pAAV/HBV1.2_C2_ was detected using a HBsAg diagnostic kit (S10980090, Wantai, China).

### 2.9 LGALS3 gene knockdown

pAAV/HBV1.2 or pAAV-MCS were transfected using Lipo8000™ transfection reagent (C0533, Beyotime, China) into HEPG2 and HUH7 cells in six-well plates. After 24 h, the transfection medium was replaced with the culture medium. Lipo8000™ transfection reagent was used to transfect siRNA with LGALS3 knockdown (si-LGALS3) or negative control (si-NC) (General, Anhui, China) into HEPG2^#^ and HUH7^#^. After 48h, supernatants and cells were collected. Cells were added to 0.5 mL Seven RNAkey™ Reagent (SM129-02, Seven, China), frozen at -80°C, and stored until further use. siRNA sequences are listed in **Supplementary Table 4**.

### 2.10 RT-qPCR

**Supplementary Table 4** presents the Primer sequences. Total RNA was extracted from cells using Seven RNAkey™ Reagent (SM129-02, Seven, China), and cDNA was synthesized using a reverse transcription kit (SM131-02A, Seven, China) according to the manufacturer’s instructions. The SYBR Green Master Mix kit (SM133-02, Seven, China) and an RT-qPCR instrument (SLAN-96p, Shanghai Hongshi, China) were used for RT-qPCR. Additionally, GAPDH was used as an internal control, and quantification was based on the 2-ΔΔCt method.

### 2.11 CCK8 assay

The CCK8 assay kit (SC119-01, Seven, China) was used to detect the proliferation of HBV-HCC cells after si-LGALS3 transfection. Cells (1× 10^5^ cells/well) were cultured with DMEM medium containing 10% fetal bovine serum. Additionally, after culture at 37°C for 24 h, 10 μL CCK8 was added to each well. Subsequently, cells were incubated at 37°C for 1 h, and absorbance was measured at 450 nm.

### 2.12 Transwell assay

Transwell chambers were used to determine the migration efficiency of the HBV-HCC cells after si-LGALS3 transfection. Cells (2 × 10^4^ cells/well) were added to the upper chamber with serum-free DMEM medium (aperture 8.0 μm), with lower chamber containing DMEM supplemented with 20% fetal bovine serum. After culturing at 37°C for 24 h, the upper chamber cells were wiped with cotton swabs, the cells that passed through the membrane were fixed with 4% paraformaldehyde, stained with 0.1% crystal violet, and then washed with PBS thrice. Finally, the migrating cells in five random fields were counted using an optical microscope.

### 2.13 Statistical analysis

R (version 4.1.2), GraphPad Prism (version 8.0) and SPSS statistical software (version 25.0.0) were used for statistical analysis. Mann–Whitney U test was used for comparison between the two groups. Kruskal-Wallis H test was used to compare multiple groups. Spearman correlation statistics was used to test the association. *p* < 0.05 was considered statistically significant, with the symbols ***, **, *, and ns representing *p* < 0.001, *p* < 0.01, *p* < 0.05, and not significant, respectively.

## 3 Results

### 3.1 Identification and functional enrichment analysis of DENRGs

**Figure 2:**
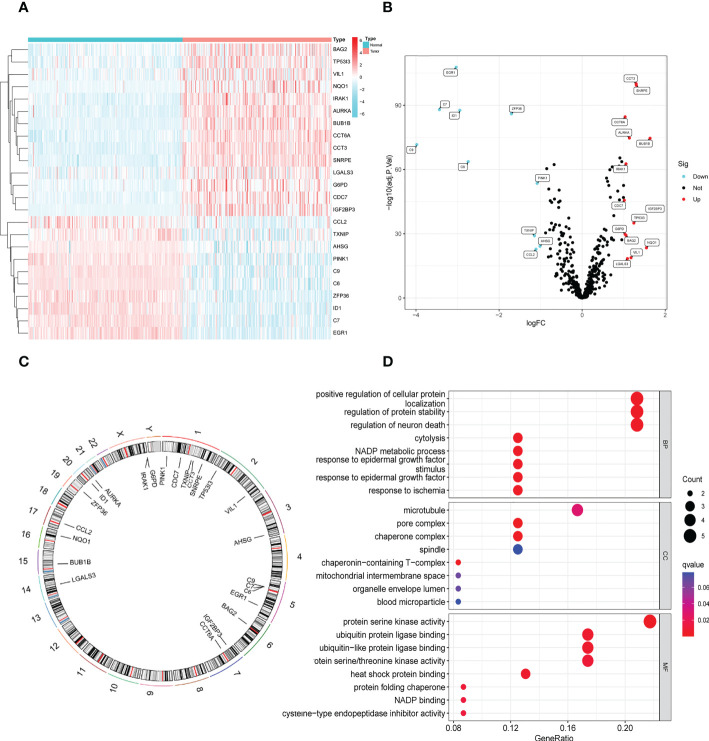
Identification and functional enrichment analysis of DENRGs. **(A)** The heatmap of 24 DENRGs in tumor and normal tissues. **(B)** The volcano plots of 24 DENRGs in tumor and normal tissues. **(C)** Chromosome schema maps of 24 DENRGs. **(D)** The GO enrichment analysis of the DENRGs.

In the GSE14520 dataset, we identified 24 DENRGs in tumor and adjacent non-tumor tissues. The expression levels of C9, C7, EGR1, ID1, C6, ZFP36, TXNIP, CCL2, PINK1, and AHSG were significantly higher in adjacent non-tumor tissues than those in tumor tissues. The expression levels of CDC7, G6PD, CCT6A, BAG2, IRAK1, LGALS3, AURKA, VIL1, TP53I3, CCT3, SNRPE, NQO1, BUB1B, and IGF2BP3 in normal tissues were significantly lower than those in tumor tissues (**Figures 2A, B**). **Figure 2C** shows the chromosomal localization of the 24 DENRGs. To explore the role of DENRGs in HBV-HCC, we performed gene ontology pathway enrichment analysis on the 24 DENRGs. Particularly, gene ontology analysis was used to determine the 24 DENRGs enrichment pathways in HBV-HCC in terms of biological process, cellular component, and molecular function. The results showed positive regulation of cellular protein localization, protein stability, neuronal death, and enhanced protein serine kinase activity (**Figure 2D**).

### 3.2 Development and validation of the NRGPS system

**Figure 3:**
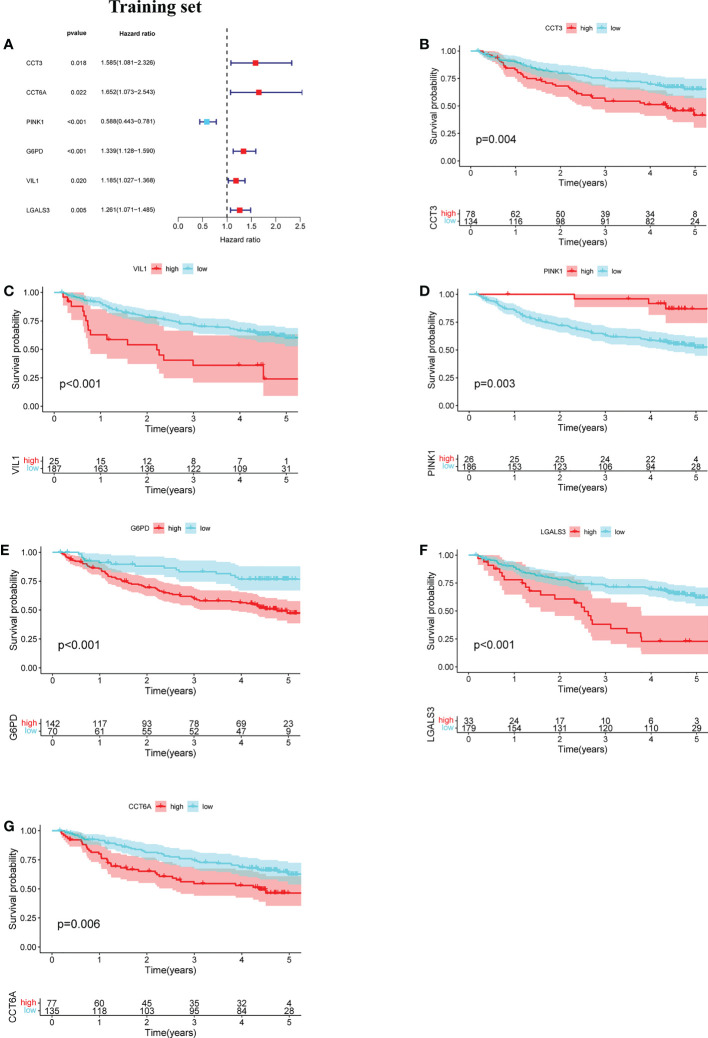
Development of the NRGPS System. **(A)** The forest map of 6 prognostic genes were obtained by Univariable Cox regression analysis. **(B–G)** Kaplan-Meier curve of 6 prognostic genes.

**Figure 4:**
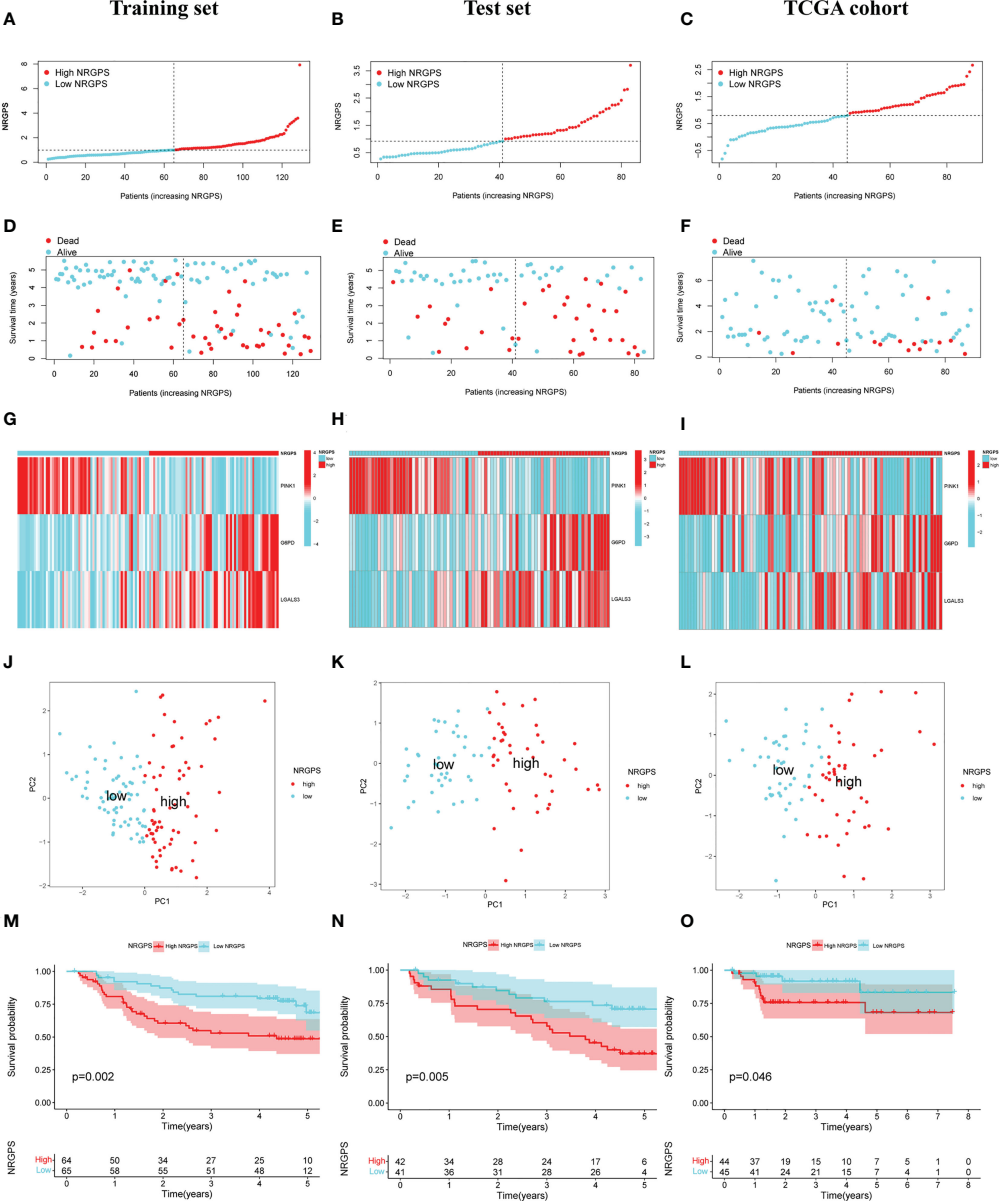
Validation of the NRGPS System. **(A–C)** The graphs of Training set, Test set and TCGA cohort. **(D–F)** The scatter plots of Training, Test set and TCGA cohort. **(G–I)** The expression of 3 DENRGs in high and low NRGPS. **(J–L)** PCA analysis in Training set, Test set and TCGA cohort. **(M–O)** Kaplan-meier survival analysis in Training set, Test set and TCGA cohort. NRGPS, necroptosis-related gene prognostic score; DENRGs, differentially expressed necroptosis-related genes.

**Figure 5:**
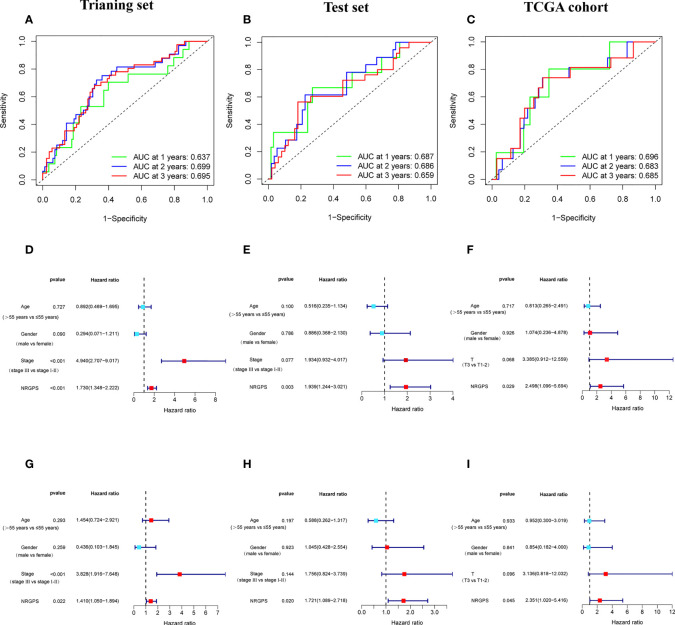
Validation of the NRGPS System. **(A–C)** The ROC curve of Training set, Test set and TCGA cohort verifies the prediction ability of this prediction model. **(D–I)** The Univariable and Multivariable COX regression analyses results of NRGPS.

The prognostic value of the 24 candidate DENRGs was investigated by conducting univariable Cox regression analysis on 129 patients with HBV-HCC in the training set. The results showed that CCT3, CCT6A, VIL1, PINK1, LGALS3, and G6PD significantly correlated with HBV-HCC prognosis (**Figures 3A–G**). Multivariable Cox regression analysis was performed for the above mentioned six genes, and the NRGPS estimated using PINK1, LGALS3, and G6PD was obtained according to the following formula: NRGPS = (-0.4025× PINK1 expression)+ (0.2081× LGALS3 expression)+ (0.2371× G6PD expression). Our results revealed that higher NRGPS scores were associated with higher risk, higher mortality, and shorter survival time in the training, test, and TCGA cohorts (**Figures 4A–F**). The heat map shows that G6PD and LGALS3 were highly expressed in the high NRGPS group, and PINK1 was highly expressed in the low NRGPS group (**Figures 4G–I**). In the principal component analysis, patients with different NRGPS scores could be clearly divided into two clusters (**Figures 4J–L**). Kaplan–Meier curves showed that the overall survival of the high-NRGPS group was shorter than that of the low-NRGPS group (**Figures 4M–O**). In the time-dependent receiver operating characteristic analysis, the areas under the 1-year curve of the training, test, and TCGA cohorts were 0.637, 0.687, and 0.696, respectively. Additionally, the 2-year area under the curve were 0.699, 0.686, and 0.683, respectively, and those of the 3-year area under the curve were 0.695, 0.659, and 0.685, respectively (**Figures 5A–C**). These results indicated that the NRGPS has high specificity and sensitivity for predicting the prognosis of HBV-HCC.

### 3.3 Independent prognostic analysis of NRGPS and GSEA

**Figure 6:**
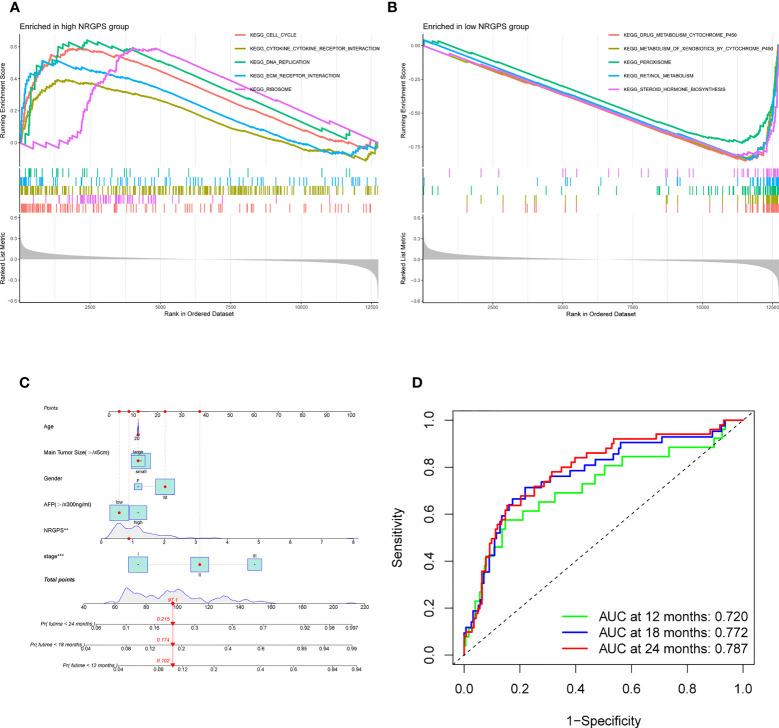
GSEA analysis and Nomogram. **(A)** GSEA in the high-NRGPS group. **(B)** GSEA in the low-NGPS group. **(C)** Nomogram. **(D)** The ROC curve of Nomogram.

This study evaluated whether NRGPS can be an independent prognostic indicator. Univariable and multivariable Cox regression analyses were performed to determine the associations between prognosis and age, gender, TNM stage, and NRGPS. Similarly, the correlation between age, gender, T stage, and NRGPS was analyzed in TCGA cohort. Univariable Cox regression analysis showed that NRGPS was correlated with patient prognosis in the GSE14520 and TCGA cohorts (**Figures 5D–F**). Multivariable Cox regression analysis confirmed that the NRGPS was an independent predictor of survival after adjusting for other clinical confounding factors in the GSE14520 and TCGA cohorts (**Figures 5G–I**). The Kyoto Encyclopedia of Genes and Genomes sets from the high- and low-NRGPS groups were used for GSEA to investigate the differences in biological characteristics between the two groups. High NRGPS enriched pathways included “cell cycle,” “spliceosome,” “ecm receptor interaction,” “DNA replication,” and “ribosome.” The enriched pathways in the low NRGPS group were “metabolism of cytochrome P450,” “metabolism of cytochrome P450 xenogeneic organisms,” “retinol metabolism,” “complement,” “steroid hormone biosynthesis,” “valine leucine,” and “isoleucine degradation” (**Figures 6A, B**). These results suggest that high NRGPS expression is associated with tumor progression and metastasis. We combined the NRGPS with clinical variables to construct a nomogram to be more suitable for clinical application. Receiver operating characteristic curves showed that the nomogram had a good predictive performance for the 1-, 1.5-, and 2-year overall survival of patients with HBV-HCC (**Figures 6C, D**).

### 3.4 Applicability of the HCC model in HBV-HCC data

The results showed that the prognostic model for HCC constructed using the available literature did not predict the survival time of patients with HBV-HCC (**Figure S1**). In contrast, the NRGPS had a better predictive ability.

### 3.5 Drug sensitivity analysis

The differences in drug sensitivity between high and low NRGPS and the two- and three-dimensional structures of the drugs are shown in **Figure S2**.

### 3.6 Analysis of immune microenvironment

**Figure 7:**
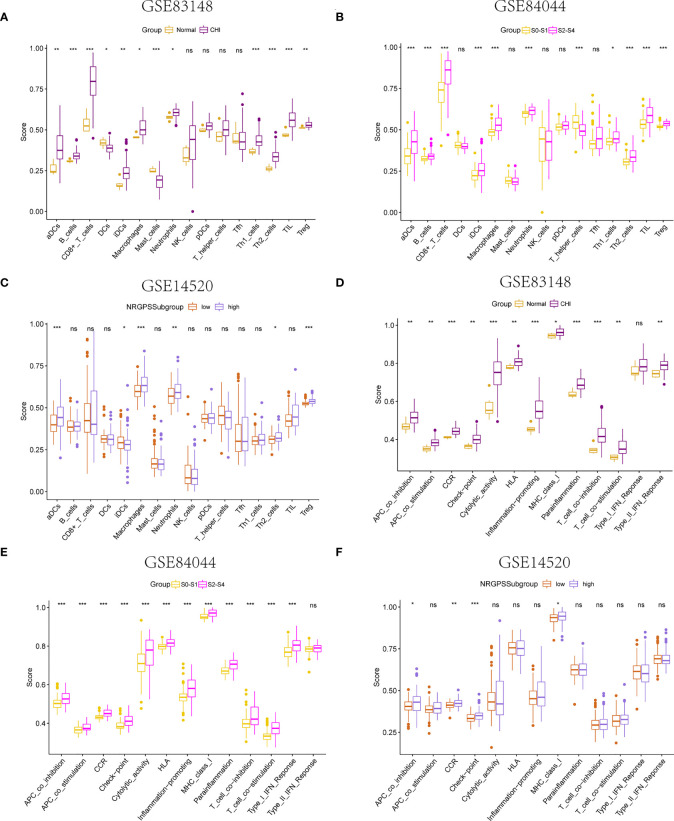
Immune microenvironment analysis. The boxplot of 16 immune cell differences in **(A)** GSE83148, **(B)** GSE84044, **(C)** GSE14520. The boxplot of 13 immune signaling pathway differences in **(D)** GSE83148, **(E)** GSE84044, **(F)** GSE14520. *P <0.05, **P <0.01, ***P < 0.001, ns, not significant

Using the ssGSEA algorithm, the infiltrating states of 16 types of immune cells and the activities of 13 types of immune-related functions in the GSE83148, GSE84044, GSE14520, and TCGA cohorts were studied. The results showed that regulatory T cells (Tregs), activated dendritic cells, immature dendritic cells, macrophages, Th2 cells, and neutrophils were significantly increased in patients with chronic HBV infection, and high fibrosis score and NRGPS were observed in the GSE83148, GSE84044, and GSE14520 cohorts (**Figures 7A–C**). The same trend was observed for immune checkpoints and cytokine-cytokine receptors (CCR) (**Figures 7D–F**). High expression of Tregs, macrophages, and Th2 cells are often associated with a poor tumor prognosis. We also analyzed the relationship between infiltrating immune cells and the immune function of the three model genes in the immune microenvironment of the GSE83148, GSE84044, GSE14520, and TCGA cohorts. The results showed that LGALS3 was more associated with increased infiltration of immune cells and expression of immune pathway compared to G6PD and PINK1.

**Figure 8:**
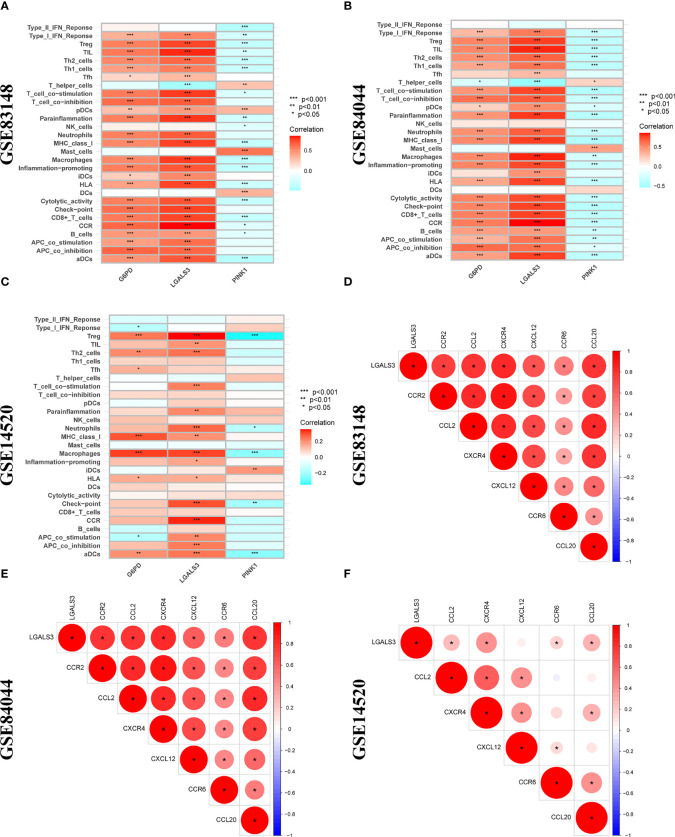
Correlation analysis between 3 DENRGs and immune microenvironment. The relationship between 3 DENRGs and immune microenvironment in **(A)** GSE83148, **(B)** GSE84044, **(C)** GSE14520. Correlation analysis of 3 DENRGs with chemokines and chemokine receptors in **(D)** GSE83148, **(E)** GSE84044, **(F)** GSE14520.

Furthermore, LGALS3 expression was significantly and positively correlated with the expression of CCR and immune checkpoint genes, as well as Treg cell infiltration (**Figures 8A–C**). As important components of the CCR, chemokines and chemokine receptors regulate cell migration, adhesion, localization, and intercellular interactions and are highly involved in tumor development. Among them, CCR2-CCL2, CXCR4-CXCL12, and CCR6-CCL20, three pairs of chemokines and their receptors, contribute to forming the inhibitory immune microenvironment and have an obvious effect on promoting tumor progression. Further analysis of the correlation between LGALS3 and chemokines and their receptors showed that LGALS3 expression was significantly and positively correlated with the expression of CCR6 and CCL20 in the CHI, HBV-HF, and HBV-HCC microenvironments (**Figures 8D–F**). **Figure S3** shows analysis of the TCGA cohort immune microenvironment.

### 3.7 Changes in model genes with disease progression

**Figure 9:**
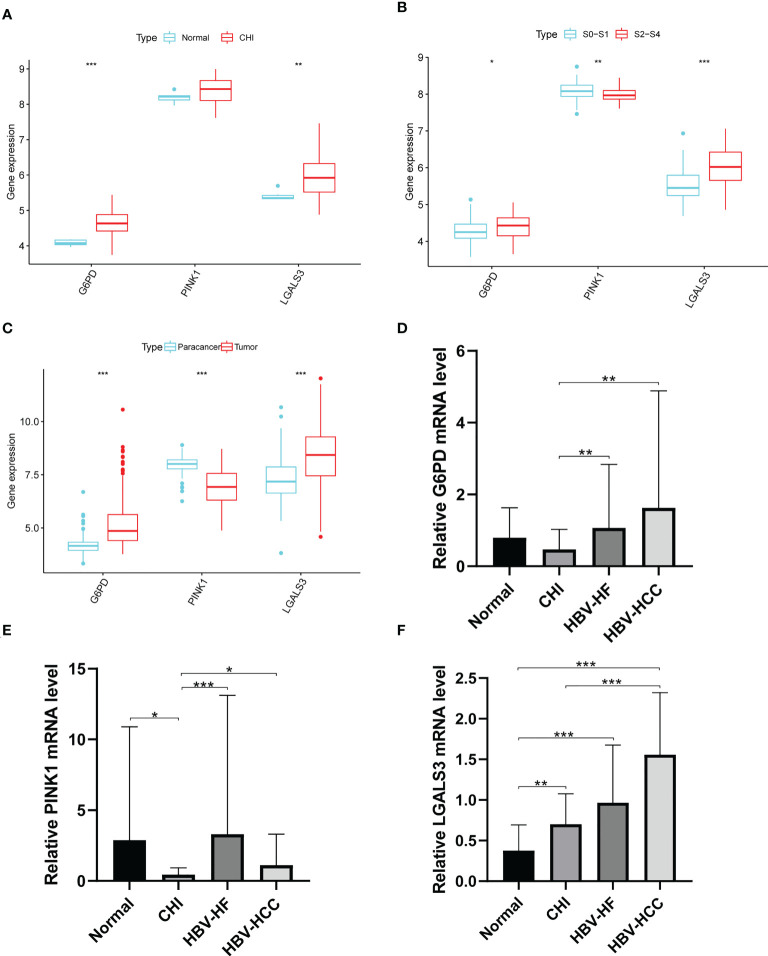
Expression of G6PD,PINK1 and LGALS3 in disease progression. Expression of G6PD, PINK1 and LGALS3 in **(A)** GSE83148, **(B)** GSE84044 and **(C)** GSE14520. **(D–F)**, The expression of G6PD, PINK1 and LGALS3 in PBMCs. *P <0.05, **P <0.01, ***P < 0.001, ns, not significant.

LGALS3 and G6PD were highly expressed in the CHI, S2-S4, and tumor groups, and PINK1 was highly expressed in the normal, S0-S1 and paracancerous tissues (**Figures 9A–C**). Among the PBMCs, PINK1 was the most expressed in the normal group (**Figure 9D**). Compared with the normal group, LGALS3 and G6PD were significantly upregulated in all stages of HBV infection (**Figures 9E, F**).

### 3.8 Expression of CCR6 and FOXP3 in PBMCs in different patients

The expression of CCR6 and FOXP3 genes was significantly increased after HBV infection (**Figures S4A–B**); however, there was no significant correlation with the expression of LGALS3 in PBMCs (**Figures S4C–H**).

### 3.9 Analysis of transfection efficiency

The transfection efficiency of HEPG2 was 61.38 ± 2.44% and that of HUH7 was 81.85 ± 2.68% (**Figure S5A**). ELISA results showed that HEPG2 and HUH7 cells produced HBsAg after transfection with pAAV/HBV1.2_C2_ (**Figure S5B**).

### 3.10 Verification of gene expression

**Figure 10:**
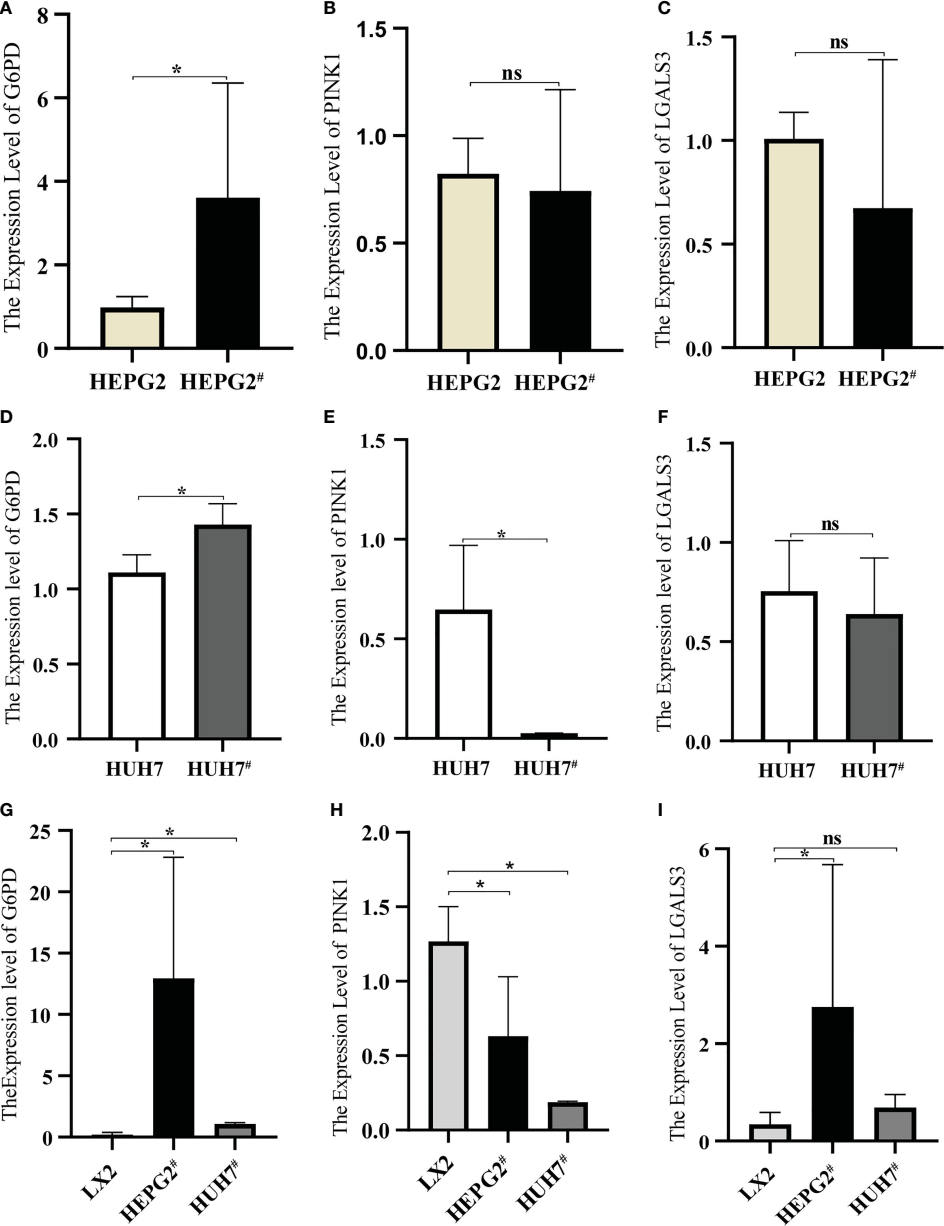
Expression of model genes in hepatocellular carcinoma cell lines. **(A)** Expression of G6PD in HEPG2 and HEPG2^#^. **(B)** Expression of PINK1in HEPG2 and HEPG2^#^. **(C)** Expression of LGALS3 in HEPG2 and HEPG2^#^. **(D)** Expression of G6PD in HUH7 and HUH7^#^. **(E)** Expression of PINK1in HUH7 and HUH7^#^, **(F)** Expression of LGALS3 in HUH7 and HUH7^#^. **(G)** Expression of G6PD in LX2, HEPG2^#^ and HUH7^#^. **(H)** Expression of PINK1in LX2, HEPG2^#^ and HUH7^#^. **(I)** Expression of LGALS3 in LX2, HEPG2^#^ and HUH7^#^. *P <0.05, ns, not significant.

qRT-PCR was used to detect the expression of each gene in the cells. After transfection with pAAV-MCV and pAAV/HBV1.2_C2_, G6PD expression in HEPG2 was significantly increased, whereas PINK1 and LGALS3 expression showed no significant changes (**Figures 10A–C**). Compared to pAAV-MCV plasmid transfection, the expression of the G6PD gene was increased, and PINK1 gene expression was decreased in HUH7 cells transfected with pAAV/HBV1.2_C2_; however, there was no significant difference in LGALS3 expression (**Figures 10D–F**). The expression level of the G6PD gene in HEPG2^#^ and HUH7^#^ was significantly higher than that in LX2 (**Figure 10G**), the expression level of the PINK1 gene in LX2 was significantly higher than that in HEPG2^#^ and HUH7^#^ (**Figure 10H**), and the expression level of the LGALS3 gene in HEPG2^#^ was significantly higher than that in LX2 (**Figure 10I**).

### 3.11 Analysis of *in vitro* LGALS3 function

**Figure 11:**
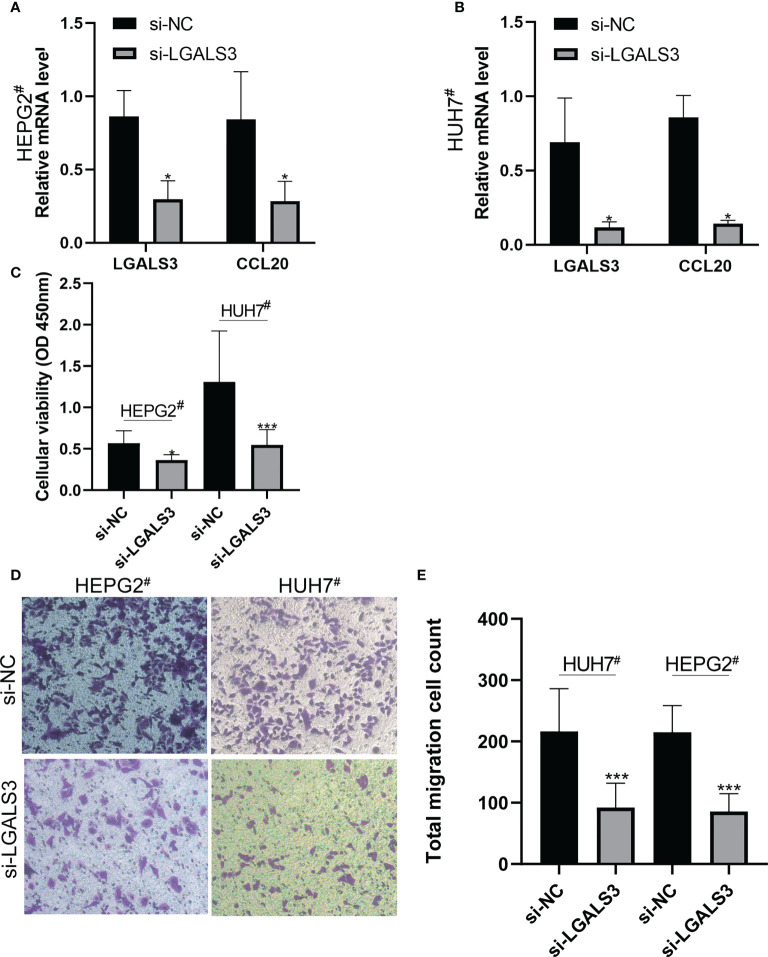
LGALS3 knockdown affects the proliferation and migration of hepatocellular carcinoma cells and the expression of CCL20. **(A)** Changes of CCL20 before and after LGALS3 knockdown in HEPG2^#^. **(B)** Changes of CCL20 before and after LGALS3 knockdown in HUH7^#^. **(C)** Cell proliferation before and after knockdown was detected by CCK8. **(D, E)** Transwell assay showed that cell migration ability decreased after LGALS3 knockdown. *P <0.05, ***P <0.001.

After LGALS3 knockdown in the HEPG2^#^ and HUH7^#^ cell lines, LGALS3 gene expression was downregulated and CCL20 expression was also significantly decreased (**Figures 11A, B**). Moreover, the results of the CCK8 cell proliferation assay showed that the proliferation of cancer cells decreased significantly after LGALS3 knockdown (**Figure 11C**), and the results of the transwell assay showed that LGALS3 knockdown significantly affected the migratory ability of cancer cells (**Figures 11D, E**).

## 4 Discussion

NRGs play a vital role in the development of HCC, and many studies have used them to establish risk-prediction models for prognosis prediction of patients with HCC (10–15). However, currently established prognostic models using NRGs for HCC are not applicable to patients with HBV-HCC, and the role of NRGs in the adverse progression of chronic HBV infection remains unclear. In this study, we systematically analyzed the expression level and correlation of NRGs in HBV-HCC and paracarcinoma tissues. Gene ontology analysis revealed 24 DENRGs that were enriched for pathways related to the proliferation and migration of tumor cells (17, 18). In addition to HBV-HCC tissues, LGALS3 expression was also upregulated in CHI tissues and tissues with high fibrosis scores. Subsequently, univariable and multivariable Cox regression analyses established a prognostic model comprising G6PD, PINK1, and LGALS3. Univariable and multivariable Cox, Kaplan–Meier, and receiver operating characteristic curve analyses all indicated that the NRGPS had good predictive performance and could be used as an independent prognostic indicator for patients with HBV-HCC. The gene expression and predictive power of the model were verified in our constructed HBV-HCC cell model and TCGA dataset.

LGALS3, also known as Galectin-3, encodes the carbohydrate-binding protein Galectin-3, which is a member of the galactoagglutinin family. It is mainly localized in the cytoplasm but is also expressed in the nucleus, cell surface, and extracellular (19). Galectin-3 expression in peripheral blood and liver is associated with the progression of chronic and acute liver failure, liver fibrosis, HCC, and other liver diseases (20–22). Moreover, our experimental data revealed persistent upregulation of the LGALS3 gene in the HBV-infected group. It has been suggested that the HBV-X protein produced by HBV in hepatocytes can transactivate the Galectin-3 promoter or upregulate Galectin-3 expression through the CREB/ATF-transcription pathway after HBV infection, and this phenomenon is more obvious in normal liver cells than in hepatoma cells (23). As reported in the literature, LGALS3 expression in HEPG2 and HUH7 cells did not change significantly after transfection with the HBV plasmid, which results from the high expression of LGALS3 in HCC cells that reduces the influence of HBV on LGALS3 expression.

The changes in the immune microenvironment are closely related to the occurrence and progression of the disease. Galectin-3 plays a role in many cellular functions, including apoptosis, innate immunity, and T-cell regulation, and is a vital component of the immune microenvironment (24, 25). HBV has multiple immunosuppressive effects, a key factor in the progression of HBV infection from chronic infection to HCC (26). In ssGSEA analysis, we found that the expression of Treg cells and immune checkpoints was upregulated in the chronic HBV infection, high fibrosis score, and high-NRGPS groups. Additionally, our population analysis results showed that the expression of FOXP3, a key transcription factor of Treg cells, was higher in the experimental group than in the control group. Tregs can inhibit the proliferation of CD8+T cells and the production of granzyme A and B and perforin, ultimately leading to a decline in the normal immune defense and surveillance functions of CD8+T cells in the microenvironment (27, 28). High infiltration of Tregs and upregulation of immune checkpoints promoted the formation of an immunosuppressive microenvironment (6, 29), which is conducive to the persistency of the HBV infection and to the escape of tumor cells from the surveillance of the immune system.

Further analysis of the immune microenvironment showed that high expression of LGALS3 was positively correlated with immune checkpoint gene expression, CCL20, CCR6, and Treg cells in patients with CHI, HBV-HF, or HBV-HCC. Chemokines are essential for immune cell transport and promote the recruitment of immune cells to immunoreactive sites during inflammation (30). Currently, it is known that CCL20 exclusively combines with CCR6 to form the CCR6-CCL20 axis, involved in regulating immune system homeostasis (31). CCL20 is a cytokine that can promote Treg cell infiltration, and the CCR6-CCL20 axis regulated Tregs migrate into the tumor microenvironment, thereby leading to tumor progression and poor prognosis in patients with HCC (32–35). Moreover, the oversecretion of CCL20 by myoblasts in cirrhotic HCC promotes the production of HCC by regulating aerobic glycolysis through the CCR6-receptor and the ERK/PKM2-signaling pathway (36). All of these highlight the important role of CCL20 in disease worsening after HBV infection. Although there was no significant correlation between LGALS3 and the expression of CCR6 and FOXP3 in PBMCs, the expression of CCL20 in HEPG2^#^ and HUH7^#^ decreased significantly after LGALS3 knockdown. LGALS3 may be involved in the formation of an immunosuppressive microenvironment by influencing the expression of CCL20, leading to adverse disease progression. In addition, LGALS3 is associated with the metabolism of HCC and lymph node metastasis, which is a key regulatory factor for tumor cell proliferation and migration (37, 38). Furthermore, the proliferation and migration of HEPG2^#^ and HUH7^#^ cells decreased significantly after the LGALS3 knockdown.

In conclusion, our findings identified the important role of a key gene, LGALS3, in disease progression after HBV persistence infection. However, this study has some limitations. First, we could not collect HBV-HCC liver tissue samples to verify the predictive power of our model. Second, the prognostic model established in this study still needs to be further verified in multicenter, large-scale clinical studies.

## 5 Conclusion

This study successfully constructed a prognostic model for HBV-HCC comprising G6PD, PINK1, and LGALS3, and analyzed the key role of LGALS3 in adverse disease progression after HBV persistence infection. Moreover, LGALS3 was demonstrated to be a potential therapeutic target for the adverse progression of HBV persistence infection.

## Data availability statement

The datasets presented in this study can be found in online repositories. The names of the repository/repositories and accession number(s) can be found in the article/**Supplementary Material**.

## Ethics statement

The studies involving human participants were reviewed and approved by Medical Ethics Committee of the Second Affiliated Hospital of Harbin Medical University. The patients/participants provided their written informed consent to participate in this study.

## Author contributions

JD and RZ designed the study. YX and KZ wrote and revised the manuscript. XJ and JL helped to perform the statistical analysis. MG and XC downloaded and collated the data. SZ was responsible for supervising the study. All authors contributed to the article and approved the submitted version.
