# Supplementary material for: The necroptosis related gene LGALS3 can be used as a biomarker for the adverse progression from chronic HBV infection to HCC
Source: Front Immunol. 2023 Apr 26;14:1142319. doi: 10.3389/fimmu.2023.1142319 (PMC10169569; doi:10.3389/fimmu.2023.1142319)
Supplement: Supplementary file 1 [file DataSheet_1.docx]

Supplementary Material

## Supplementary Figure S1

##
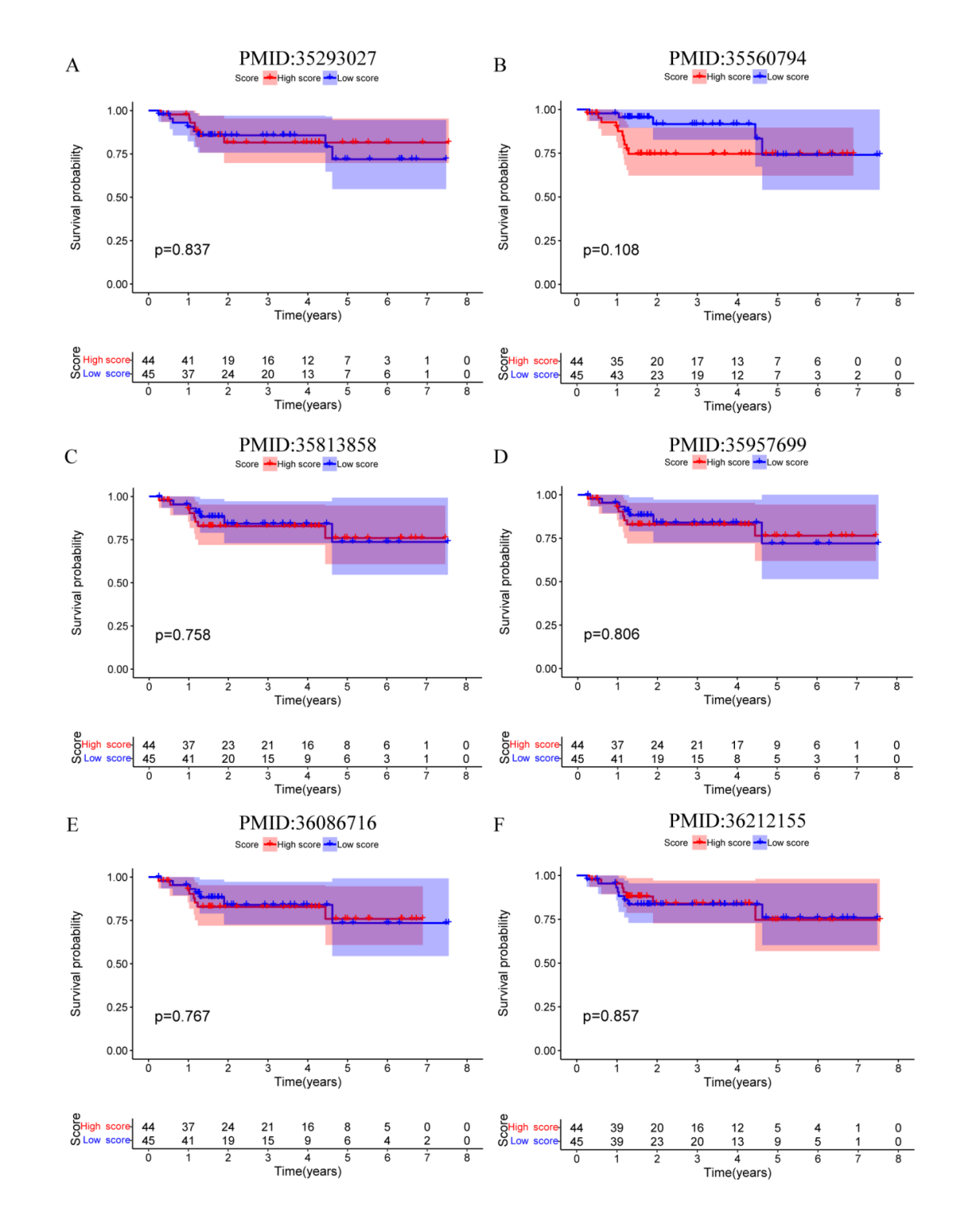


**Supplementary Figure S1 |** Applicability analysis of HCC prognostic model. (A-F), K-M analysis results of HCC prognostic model in HBV-HCC。

## Supplementary Figure S2

**
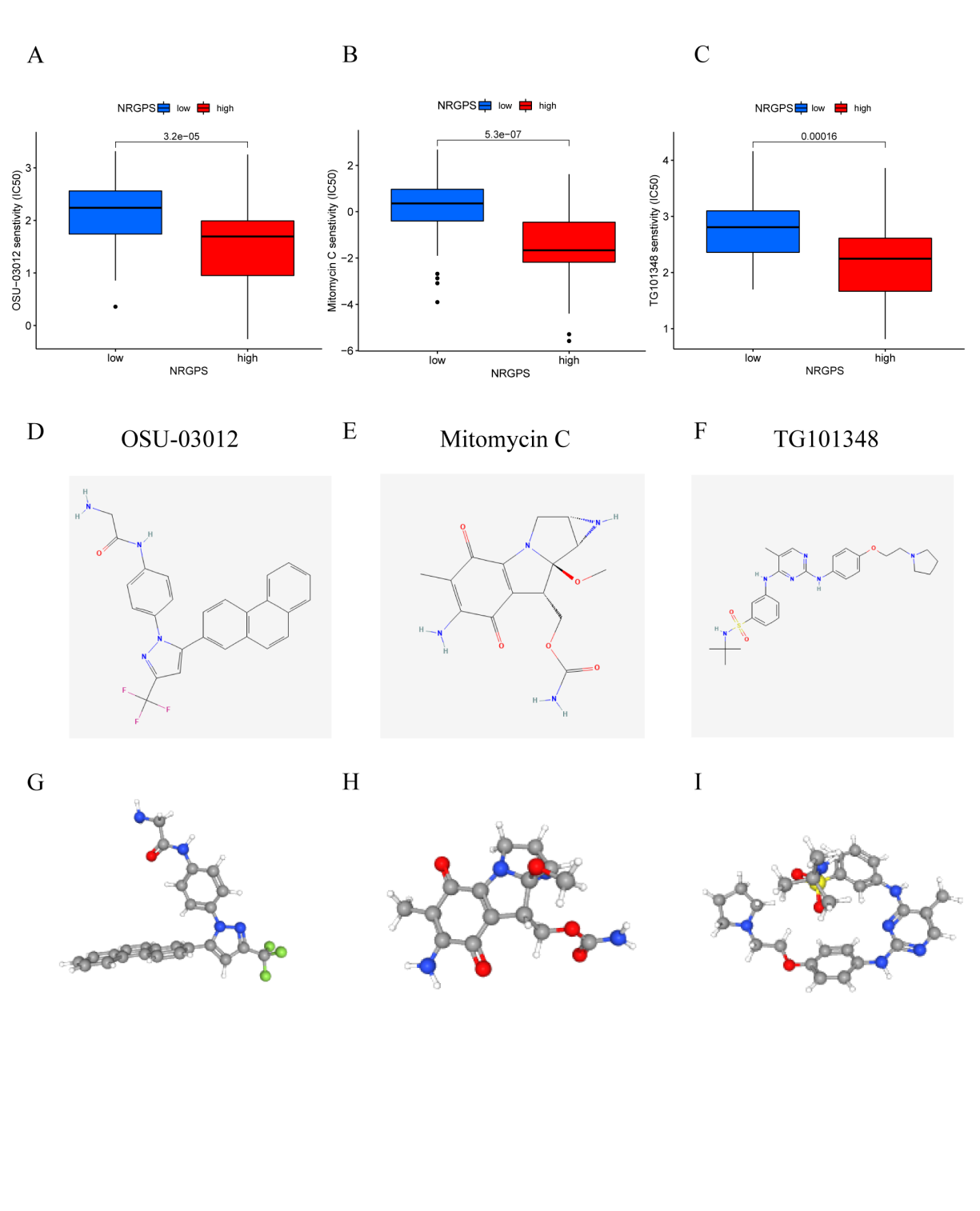
**

**Supplementary Figure S2 |** NRGPS predicts drug sensitivity. (A-C), The boxplot for predicting drug sensitivity in high and low NRGPS groups. (D-F), The 2D structure of drugs. (G-I), The 3D structure of drugs.

## Supplementary Figure S3


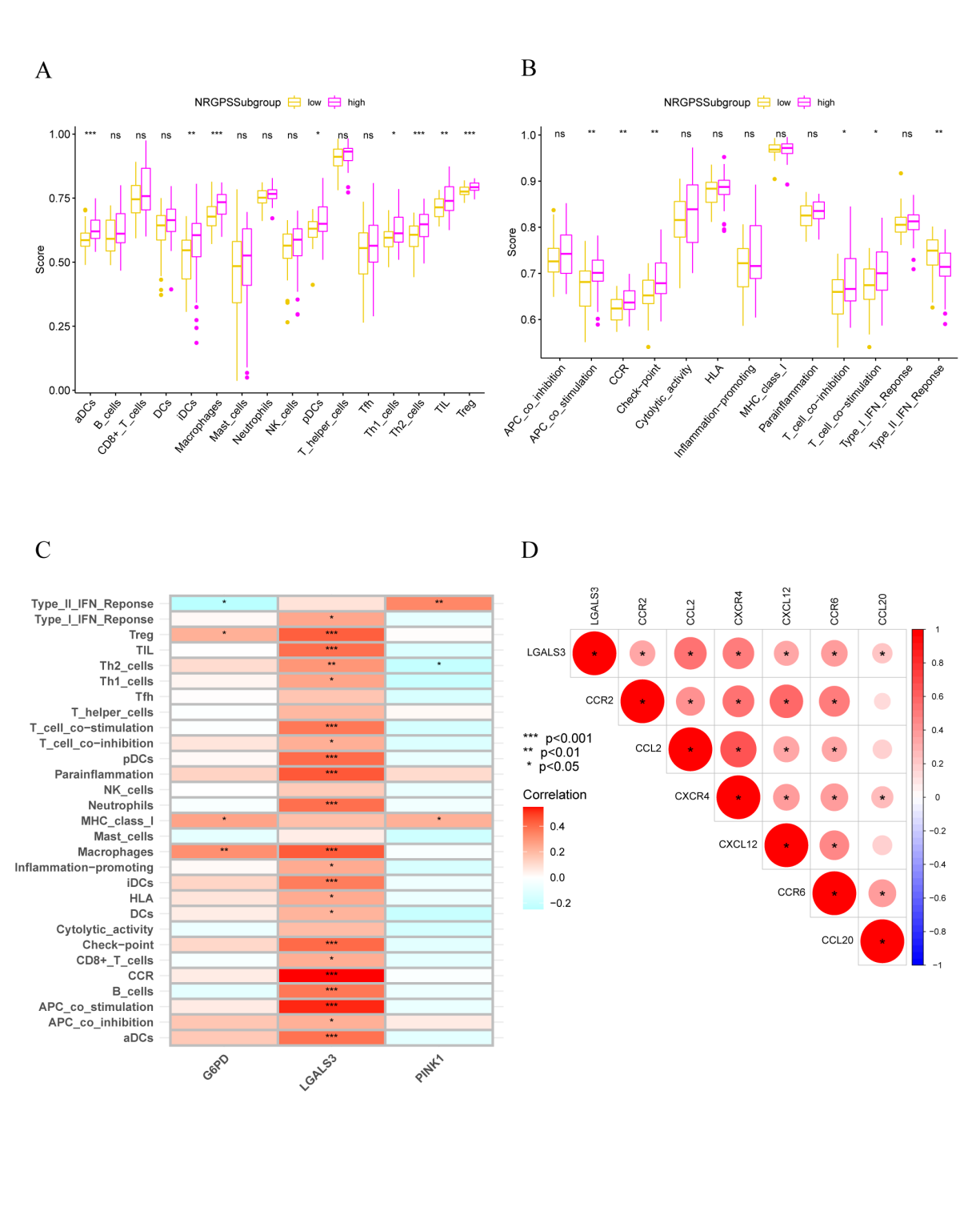


**Supplementary Figure S3 | Immune microenvironment analysis in TCGAcohort.** (A) The boxplot of 16 immune cell differences in the low-NGPS and high-NGPS groups. (B) The boxplot of 13 immune signaling pathway differences in the low-NGPS and high-NGPS groups. (C) The relationship between 3 DENRGs and immune microenvironment in TCGAcohort. (D) Correlation analysis of 3 DENRGs with chemokines and chemokine receptors in TCGAcohort.

**Supplementary Table 1**

| Variables | | GSE14520 (n =212) | | GSE84044 (n =124) | |
| --- | --- | --- | --- | --- | --- |
|  |  | Number | Percentage (%) | Number | Percentage (%) |
| Age | ≥ 55 | 71 | 33.49 | 23 | 18.55 |
|  | < 55 | 141 | 66.51 | 101 | 81.45 |
|  | Unknow | 0 | 0 | 0 | 0 |
| Gender | Male | 183 | 86.32 | 88 | 70.97 |
|  | Female | 29 | 13.68 | 36 | 29.03 |
|  | Unknow | 0 | 0 | 0 | 0 |
| Grade | G1 | 0 | 0 | 0 | 0 |
|  | G2 | 0 | 0 | 0 | 0 |
|  | G3 | 0 | 0 | 0 | 0 |
|  | G4 | 0 | 0 | 0 | 0 |
|  | Unknow | 212 | 100 | 124 | 100 |
| Clinical stage | Stage I | 89 | 41.98 | 0 | 0 |
|  | Stage II | 76 | 35.85 | 0 | 0 |
|  | Stage III | 47 | 22.17 | 0 | 0 |
|  | Stage IV | 0 | 0 | 0 | 0 |
|  | Unknow | 0 | 0 | 0 | 0 |
| positive HBsAg or serum HBV-DNA | Positive | 212 | 100 | 124 | 100 |
|  | Negative | 0 | 0 | 0 | 0 |
|  | Unknow | 0 | 0 | 0 | 0 |
| Tstage | T1 | 0 | 0 | 0 | 0 |
|  | T2 | 0 | 0 | 0 | 0 |
|  | T3 | 0 | 0 | 0 | 0 |
|  | T4 | 0 | 0 | 0 | 0 |
|  | Unknow | 0 | 0 | 0 | 0 |
| Mstage | M0 | 0 | 0 | 0 | 0 |
|  | M1 | 0 | 0 | 0 | 0 |
|  | Unknow | 0 | 0 | 0 | 0 |
| Nstage | N0 | 0 | 0 | 0 | 0 |
|  | N1 | 0 | 0 | 0 | 0 |
|  | N2 | 0 | 0 | 0 | 0 |
|  | N3 | 0 | 0 | 0 | 0 |
|  | unknow | 0 | 0 | 0 | 0 |

| Variables | | GSE83148 (n = 128 ) | | TCGA (n = 89) | |
| --- | --- | --- | --- | --- | --- |
|  |  | Number | Percentage (%) | Number | Percentage (%) |
| Age | ≥ 55 | 0 | 0 | 42 | 47.19 |
|  | < 55 | 0 | 0 | 47 | 52.81 |
|  | Unknow | 128 | 100 | 0 | 0 |
| Gender | Male | 0 | 0 | 14 | 15.73 |
|  | Female | 0 | 0 | 75 | 84.27 |
|  | Unknow | 128 | 100 | 0 | 0 |
| Grade | G1 | 0 | 0 | 4 | 4.49 |
|  | G2 | 0 | 0 | 31 | 34.83 |
|  | G3 | 0 | 0 | 45 | 50.56 |
|  | G4 | 0 | 0 | 9 | 10.11 |
|  | Unknow | 128 | 100 | 0 | 0 |
| Clinical stage | Stage I | 0 | 0 | 60 | 67.42 |
|  | Stage II | 0 | 0 | 18 | 20.22 |
|  | Stage III | 0 | 0 | 8 | 8.98876 |
|  | Stage IV | 0 | 0 | 1 | 1.12 |
|  | Unknow | 0 | 0 | 2 | 2.24 |
| positive HBsAg or serum HBV-DNA | Positive | 122 | 95.31 | 89 | 100 |
|  | Negative | 6 | 4.69 | 0 | 0 |
|  | Unknow | 0 | 0 | 0 | 0 |
| Tstage | T1 | 0 | 0 | 61 | 68.54 |
|  | T2 | 0 | 0 | 18 | 20.22 |
|  | T3 | 0 | 0 | 8 | 8.99 |
|  | T4 | 0 | 0 | 2 | 2.25 |
|  | Unknow | 0 | 0 | 0 | 0 |
| Mstage | M0 | 0 | 0 | 79 | 88.76 |
|  | M1 | 0 | 0 | 1 | 1.12 |
|  | Unknow | 0 | 0 | 9 | 10.11 |
| Nstage | N0 | 0 | 0 | 82 | 92.13 |
|  | N1 | 0 | 0 | 0 | 0 |
|  | N2 | 0 | 0 | 0 | 0 |
|  | N3 | 0 | 0 | 0 | 0 |
|  | unknow | 0 | 0 | 7 | 7.87 |

**Supplementary Table 2**

| **Inclusion criteria** |  |
| --- | --- |
| Chronic HBV infected | HBsAg positive for more than 6 months |
| HBV-associated cirrhosis | The clinical diagnosis was cirrhosis with HBV infection |
| HBV-associated liver cancer | The clinical diagnosis was hepatocellular carcinoma with HBV infection |
| Control group | History of HBV vaccination, only HBsAb positive and other physical indicators are normal |
| Additional condition | Excluding other types of viral infections (including hepatitis A, C, D, and E viruses), alcohol,  autoimmune, and other diseases that can cause chronic liver injury. |

## **Supplementary Table 3**

| Variables | | Normal (n = 31) | | CHI (n = 30) | | HBV-HF (n = 21) | |
| --- | --- | --- | --- | --- | --- | --- | --- |
|  |  | Number | Percentage(%) | Number | Percentage(%) | Number | Percentage(%) |
| Age | ≥ 55 | 9 | 29.03 | 6 | 20.00 | 7 | 33.33333333 |
|  | < 55 | 22 | 70.97 | 24 | 80.00 | 16 | 76.19047619 |
|  | Unknow | 0 | 0 | 0 | 0.00 | 0 | 0 |
| Gender | Male | 20 | 64.5 | 14 | 46.67 | 12 | 57.77 |
|  | Female | 11 | 35.5 | 16 | 53.33 | 9 | 42.23 |
| HBsAg/  HBV DNA | positive | 0 | 0 | 0 | 0.00 | 21 | 100 |
|  | negative | 0 | 0 | 30 | 100.00 | 0 | 0 |
|  | Unknow | 31 | 100 | 0 | 0.00 | 0 | 0 |
| HBsAb | positive | 31 | 100 | 0 | 0.00 | 0 | 0 |
|  | negative | 0 | 0 | 0 | 0.00 | 0 | 0 |
|  | Unknow | 0 | 0 | 30 | 100.00 | 21 | 100 |
| ALT | ≥ 40 | 2 | 6.45 | 16 | 53.33 | 5 | 23.80952381 |
|  | < 40 | 26 | 83.87 | 12 | 40.00 | 14 | 66.66666667 |
|  | Unknow | 3 | 9.68 | 2 | 6.67 | 2 | 9.523809524 |
| AST | ≥ 35 | 1 | 3.23 | 17 | 56.67 | 6 | 28.57142857 |
|  | < 35 | 27 | 87.10 | 11 | 36.67 | 13 | 61.9047619 |
|  | Unknow | 3 | 9.68 | 2 | 6.67 | 2 | 9.523809524 |
|  |  |  |  |  |  |  |  |
|  | HBV-HCC (n = 20 ) | |  |  |  |  |  |
|  | Number | Percentage |  |  |  |  |  |
|  |  | (%) |  |  |  |  |  |
| Age | 7 | 23.33333333 |  |  |  |  |  |
|  | 13 | 43.33333333 |  |  |  |  |  |
|  | 0 | 0 |  |  |  |  |  |
| Gender | 12 | 40 |  |  |  |  |  |
|  | 8 | 26.66666667 |  |  |  |  |  |
| HBsAg/HBV DNA | 20 | 100 |  |  |  |  |  |
|  | 0 | 0 |  |  |  |  |  |
|  | 0 | 0 |  |  |  |  |  |
| HBsAb | 0 | 0 |  |  |  |  |  |
|  | 0 | 0 |  |  |  |  |  |
|  | 20 | 100 |  |  |  |  |  |
| ALT | 6 | 20 |  |  |  |  |  |
|  | 7 | 23.33333333 |  |  |  |  |  |
|  | 7 | 23.33333333 |  |  |  |  |  |
| AST | 9 | 30 |  |  |  |  |  |
|  | 4 | 13.33333333 |  |  |  |  |  |
|  | 7 | 23.33333333 |  |  |  |  |  |

**Supplementary Table 4**

| Primer |  | Sequences (5'to3') |
| --- | --- | --- |
| G6PD | F | CGAGGCCGTCACCAAGAAC |
|  | R | GTAGTGGTCGATGCGGTAGA |
| PINK1 | F | GCCTCATCGAGGAAAAACAGG |
|  | R | GTCTCGTGTCCAACGGGTC |
| LGALS3 | F | ATGGCAGACAATTTTTCGCTCC |
|  | R | GCCTGTCCAGGATAAGCCC |
| CCL20 | F | TGCTGTACCAAGAGTTTGCTC |
|  | R | CGCACACAGACAACTTTTTCTTT |
| CCR6 | F | TTCAGCGATGTTTTCGACTCC |
|  | R | GCAATCGGTACAAATAGCCTGG |
| FOXP3 | F | AAGGAAAGGAGGATGGACG |
|  | R | CAGGCAAGACAGTGGAAACC |
| GAPDH | F | GGAGCGAGATCCCTCCAAAAT |
|  | R | GGCTGTTGTCATACTTCTCATGG |
| si-LGALS3 | F | CAAACAGAAUUGCUUUAGATT |
|  | R | UCUAAAGCAAUUCUGUUUGTT |
| si-NC | F | UUCUCCGAACGUGUCACGUTT |
|  | R | ACGUGACACGUUCGGAGAATT |

**Supplementary Table 4 |**  The Primer sequences of G6PD, PINK1, LGALS3, CCL20, CCR6, FOXP3, GAPDH, siRNA-488 and negative contorl.

**Supplementary Figure S4**


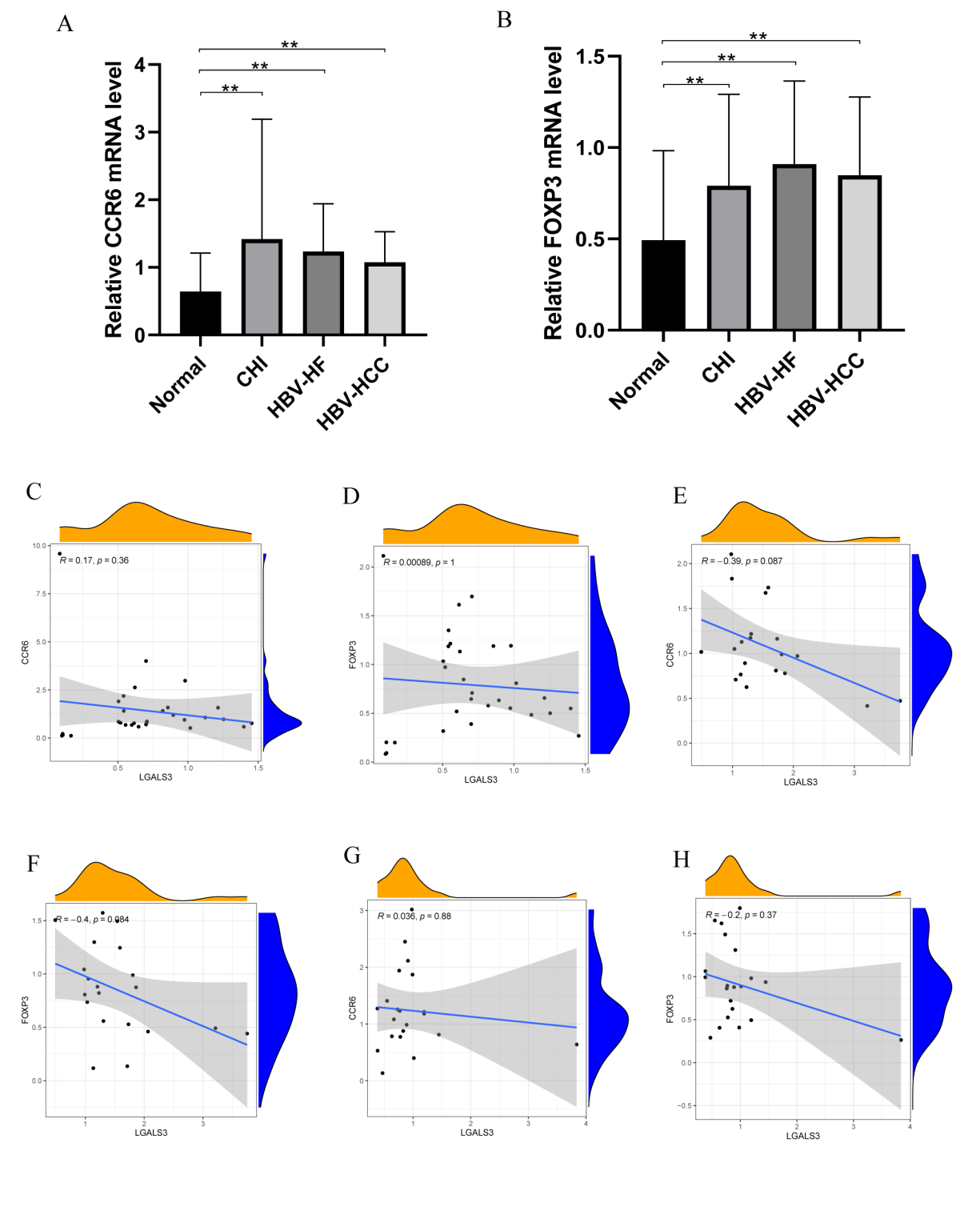


**Supplementary Figure S4 |** Expression of CCR6 and FOXP3 in disease progression. (A) Expression of CCR6 in PBMCs of different groups. (B) The expression of FOXP3 in PBMCs of different groups. (C-H) Correlation analysis of CCR6 and FOXP3 with LGALS3 in PBMCs of CHI, HBV-HF and HBV-HCC patients.

**Supplementary Figure S5**


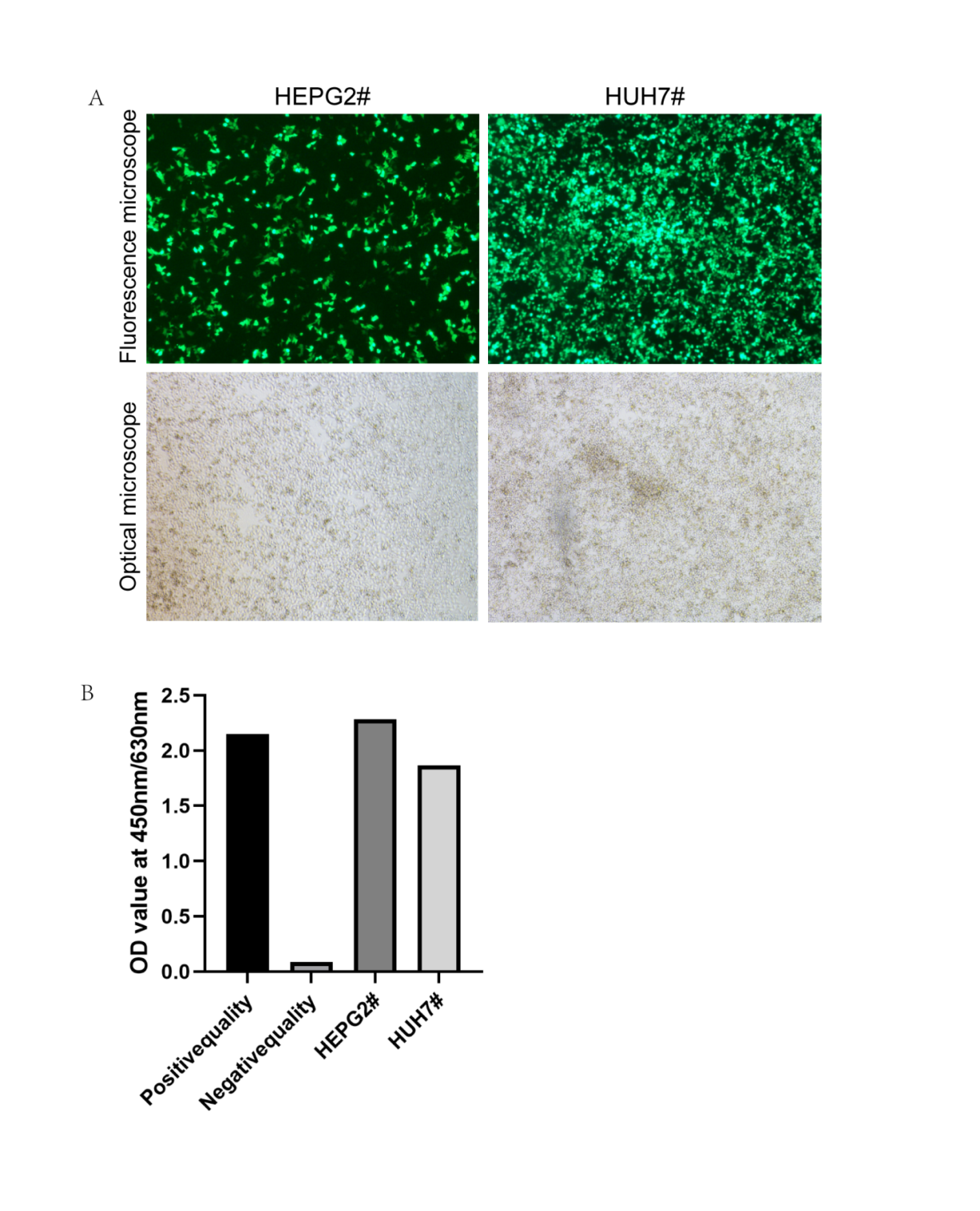


**Supplementary Figure S5 |** Fluorescence expression of pmaxGFP after co-transfection with pAAV /HBV1.2C2 for 48h (×40). A: Fluorescence and white light of HEPG2 and HUH7 after transfection for 48h. B: Expression of HBsAg 48h after co-transfection with pmaxGFP and pAAV /HBV1.2_C2_.
